# Observation of cortical state–based learning in infants in a functional near-infrared spectroscopy paradigm

**DOI:** 10.1117/1.NPh.12.2.025005

**Published:** 2025-04-05

**Authors:** Mohinish Shukla, Anna Martinez-Alvarez, Judit Gervain

**Affiliations:** a Università degli Studi di Padova, Dipartimento di Psicologia dello Sviluppo e della Socializzazione, Padua, Italy; b Università degli Studi di Padova, Padova Neuroscience Center, Padua, Italy; c CNRS and Université Paris Cité, Integrative Neuroscience and Cognition Center, Paris, France

**Keywords:** functional near-infrared spectroscopy, infant, artificial grammar learning, resting-state, state-based learning, connectivity

## Abstract

**Significance:**

Learning can be context-dependent, with better outcomes under some circumstances than others. Adult functional magnetic resonance imaging studies have shown that learning outcomes vary as a function of participants’ brain states—patterns of intrinsic neural activity—prior to the learning task. Whether this is also the case in young infants is currently unknown. We report the first functional near-infrared spectroscopy (fNIRS) study that shows prior brain state-dependent learning in a language task in 6.5-month-old infants. Babies whose functional connectivity was lower in the right hemisphere, but not in the left, during a 2-min period prior to the task learned better a grammatical regularity in an artificial grammar learning task.

**Aim:**

Adult neuroimaging studies have shown that variability in brain states immediately before specific learning tasks is correlated with variability in learning outcomes. Whether the developing infant brain also shows similar state-based learning is currently unknown.

**Approach:**

We have explored whether 6.5-month-old infants’ ability to learn artificial grammar was related to their brain state during a 2-min baseline period of rest prior to the grammar task. We have asked if functional connectivity, a global metric of the cortical brain state, as measured by fNIRS, is correlated with learning a non-adjacent regularity in the artificial grammar task.

**Results:**

We have found that the overall level of functional connectivity in the 2-min period immediately prior to the learning experience is negatively correlated with the fNIRS measure of learning in the right hemisphere but not in the left.

**Conclusions:**

We show for the first time that the cortical state of an infant immediately prior to a learning experience determines how well that infant learns and that this can account for some of the variability in learning outcomes.

## 1 Introduction

Acquiring knowledge in the real world is a messy affair, particularly for infants. Imagine a parent telling their baby that the new toy that grandma sent is a *moogey*. Whether the word *moogey* and its association with grandma’s toy registers with the baby is a function of the particular cognitive makeup of the baby herself, her previously acquired knowledge, and her current environment. For example, already possessing a similar-looking toy with a similar-sounding name would make acquisition easier,^1^ as would paying attention to the parent instead of her older brother playing across the room.^2^ Put differently, the efficacy of a learning moment presumably depends on the current state of the dynamically changing landscape of an infant’s brain/mind, with the dynamics and the current state being determined by constitutive and ephemeral factors, both internal and external.

In referring to the “state” of the brain, we intend a broad conceptualization, where we take a particular state of (part of) the brain to be a specific configuration of measured neural activity. A prominent example is the so-called resting state characterized by coordinated activity [as measured by functional magnetic resonance imaging (fMRI)] among areas that form the default mode network (DMN), such as the medial prefrontal cortex and the posterior cingulate cortex.^3^ That is, the resting state corresponds to a specific pattern of correlations between areas of the DMN. Such states need not be limited to patterns across the entire human cortex, or even to humans. As another example from rodent neuroscience, Erchova and Diamond showed that the simultaneous deflections of pairs of rat whiskers increased the co-firing (the cross-correlation in spiking activity) in specific cortical neurons corresponding to these “trained” whiskers. However, this increase in co-firing only happened when the whiskers were simultaneously deflected during periods of increased synchronic cortical activity (“bursting” states) but not in the inter-burst states.^4^

A few previous adult studies have examined the relation between brain states prior to a learning experience and the eventual learning outcome. Lewis et al.^5^ sought to understand the functional significance of spontaneous (i.e., in the absence of an overt task) neural activity as measured by fMRI. They found systematic changes in the correlation structure of such spontaneous brain activity after a visual discrimination learning task. These differences in correlation structure were predictive of the degree of learning by the participants. Subsequently, it was shown that similar differences immediately before the learning phase predict learning outcomes.^6^ Because the participants in these experiments are in a state of rest, in the sense that they are not doing a specific task, in the period immediately before the training commences, the measures of functional connectivity (correlations in activity among voxels) in this state are called resting-state fMRI (rs-fMRI). Indeed, rs-fMRI typically finds higher correlations among areas associated with the DMN.

Subsequently, such rs-fMRI experiments were used to demonstrate a predictive relationship between specific pretraining brain states and learning. In one experiment involving working memory, differences in resting brain functional connectivity patterns on a previous day were shown to predict learning outcomes on a subsequent day.^7^ In another study, Spanish-speaking participants’ resting state functional connectivity patterns were measured. Participants were subsequently trained to discriminate a phonological contrast that was not present in their native language (two Hindi consonant sounds that are not present in Spanish). The participants were subsequently tested on how well they acquired the Hindi sounds. Whether the participants were trained intensively for a single day, or trained over two weeks, variations in their initial resting brain states were predictive of their final performance in the Hindi task.^8^ These studies provided converging evidence that resting brain states can index constitutive functional brain states that predict learning in adults.

Here, we report the first study demonstrating brain state-dependent learning in young infants. As infants have less voluntary control over their cognitive resources, attentional resources, and learning processes than adults, understanding whether specific brain states are conducive to learning has important implications for theories of cognitive development as well as for education. Specifically, if it can be demonstrated that specific patterns of intrinsic brain activity before a learning experience lead to better or worse outcomes, then this paves the way to better characterizing the sources of variance that lead to observed differences in learning outcomes. Future studies can build on these observations by examining how constitutive (e.g., genetic) and context-specific (e.g., environmental, social, or otherwise) factors result in brain states linked to better or worse learning outcomes, with implications for targeted interventions that promote brain states linked to better outcomes. Our study thus contributes to a better understanding of individual variation in language learning.

Toward this end, we measured 6.5-month-old infants’ brain activity at rest and in a well-established artificial language learning paradigm^9^ using fNIRS and assessed whether functional connectivity during the period immediately prior to the learning experience predicted learning outcomes. Although we examine the period immediately prior to the learning experience, when infants are not being exposed to any particular stimuli, we remain agnostic as to whether this period corresponds to the “resting state” as defined in adult studies. That is, we are interested in the functional brain state in the period prior to the learning experience whether or not it genuinely corresponds to what is typically understood as the “resting state,” which shows specific patterns of activity in the DMN.

Previous studies using fNIRS have demonstrated that the infant brain shows evidence of extracting structure from artificial languages (see Gemignani et al.,^10^ for a recent meta-analysis). For example, 9-month-old infants are able to learn a non-adjacent regularity that holds over the first and last syllables of trisyllabic sequences (i.e., AxB, e.g., ***pe**ta**bu***, ***pe**go**bu***, ***pe**na**bu***), especially when the dependency is highlighted by prosodic cues.^9^ In this study, infants listened to blocks of artificially generated speech consisting of sets of trisyllabic sequences. In one experimental condition, half of the trisyllables followed an AxB rule, where the first syllable always predicted the third, the middle “x” syllable varied (rule condition), whereas, in the other half of the trisyllables, the same syllables constituted triplets in a randomized order, so there was no consistent relation between the syllables of the triplets (no rule condition). In addition, the A and B syllables had a higher pitch than the x syllables, resulting in a prosodic cue that highlighted the non-adjacent dependency in the rule condition but fell on random syllables in the no rule condition. Infants’ brain responses were stronger to the rule than to the no rule condition in bilateral temporal areas.

**Fig. 1:**
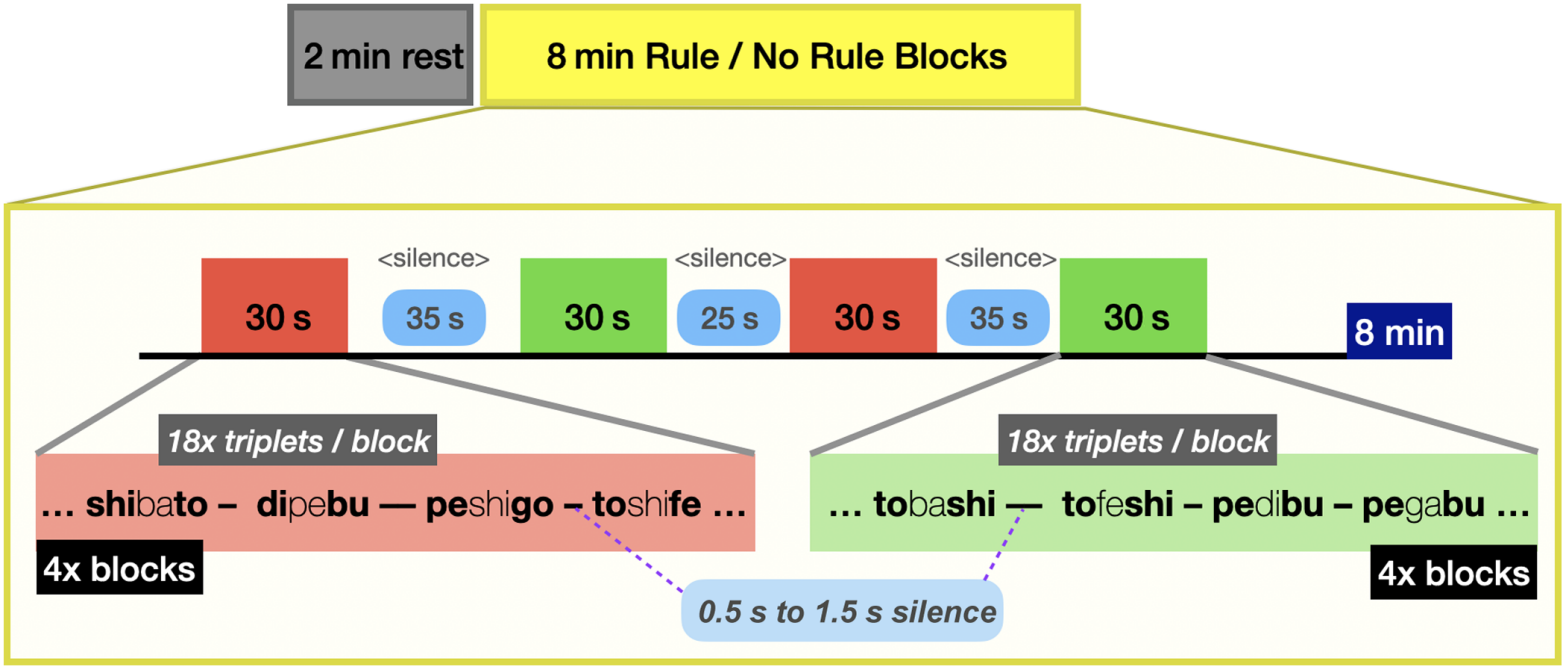
Experimental paradigm. fNIRS data were continuously gathered, first for a 2-min rest period, followed by the main learning experiment, which consisted of four blocks each of rule and no rule conditions, separated by a silent period that was randomly chosen between 25 and 35 s. Each block consisted of 18 triplets from the rule or the no rule conditions; triplets were separated by a silent period between 0.5 and 1.5 s.

**Fig. 2:**
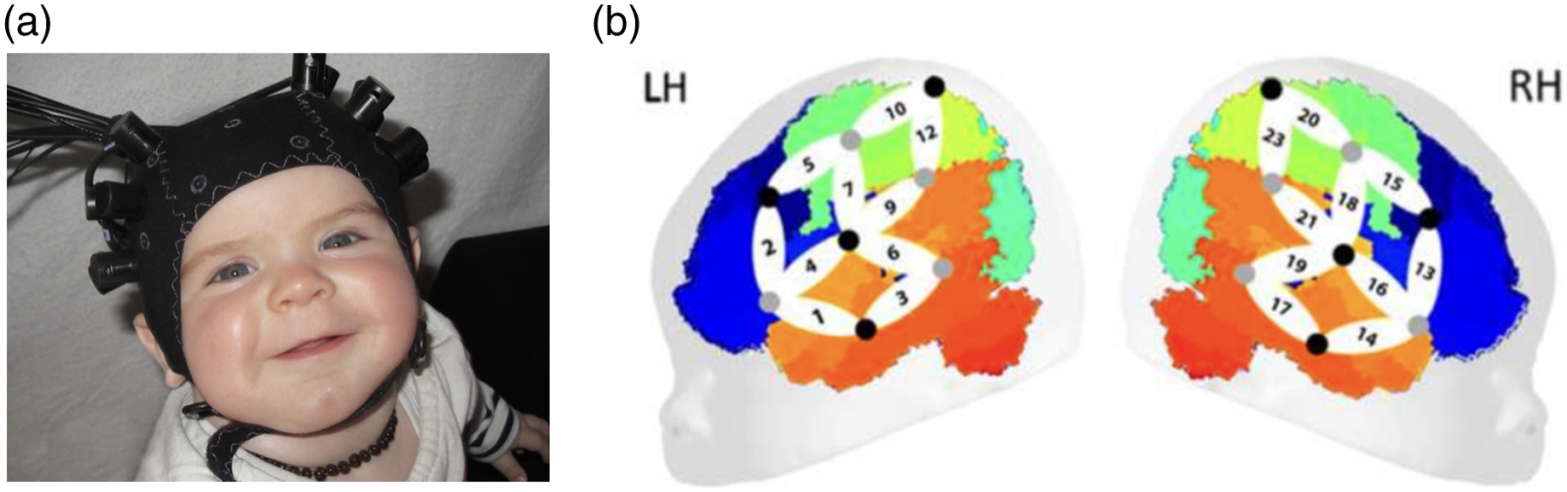
Probe layout. Panel (a) shows an infant in our study wearing the fNIRS headgear. Panel (b) shows the estimated location of channels (numbered locations) on a generic head/brain template.

The present study used this artificial grammar learning paradigm^9^ to ask whether individual variability in brain states measured for a period of 2 min prior to exposure to the artificial grammar was predictive of learning outcomes (see Fig. 1). Specifically, 6.5-month-old infants listened to rule and no rule blocks of artificially generated trisyllabic sequences as in the previous study while their brain responses were measured using fNIRS. In the rule condition, the non-adjacent dependency was implemented as both the AxB structure and increased pitch on the A and B syllables. In the no rule condition, syllables together with the pitch they carried (high pitch for A and B, low pitch for the “x” syllables) were randomized within triplets. In addition, brain activation was recorded for 2 min at rest before the artificial grammar task. Brain activation was measured using fNIRS over the bilateral temporal, parietal, and frontal brain areas, as in previous studies^9^ (see Fig. 2).

We decided to test 6.5-month-old infants because, at this age, the ability to learn non-adjacent regularities is not yet robust,^9^ individual variation among infants is thus more pronounced, and it is theoretically more relevant to understand whether brain states prior to the learning experience are related to learning outcomes. Furthermore, we chose grammar learning over other tasks as it is a particularly stringent test for the impact of brain states on learning. As many areas of language acquisition are implicit and automatic,^11^^,^^12^ we expect language learning to rely less on individual variability in attention. If brain states prior to language exposure still influence learning, that provides compelling evidence for state-based learning in early human development.

We first established that as a group, 6.5-month-old infants, 2.5 months younger than those tested previously, were able to learn the non-adjacent regularity in the artificial grammar task. We then computed the differential response to the rule over the no rule condition as an fNIRS index of learning (${\text{index}}^{\mathrm{oxyHb}}={\mathrm{oxyHb}}_{\text{Rule}}-{\mathrm{oxyHb}}_{\text{NoRule}}$), calculated functional connectivity during rest in the two hemispheres, and examined if the latter had any bearing on the former.

Our dataset is obtained from a larger study investigating the role of sleep in language learning in infants. After the testing session described above, which constitutes the dataset for the current study, infants were retested in a similar session a second time after a nap, overnight sleep, or no sleep. The results of this second session will be published separately.

## 2 Results

### 2.1 Learning the Non-adjacent Dependency

**Fig. 3:**
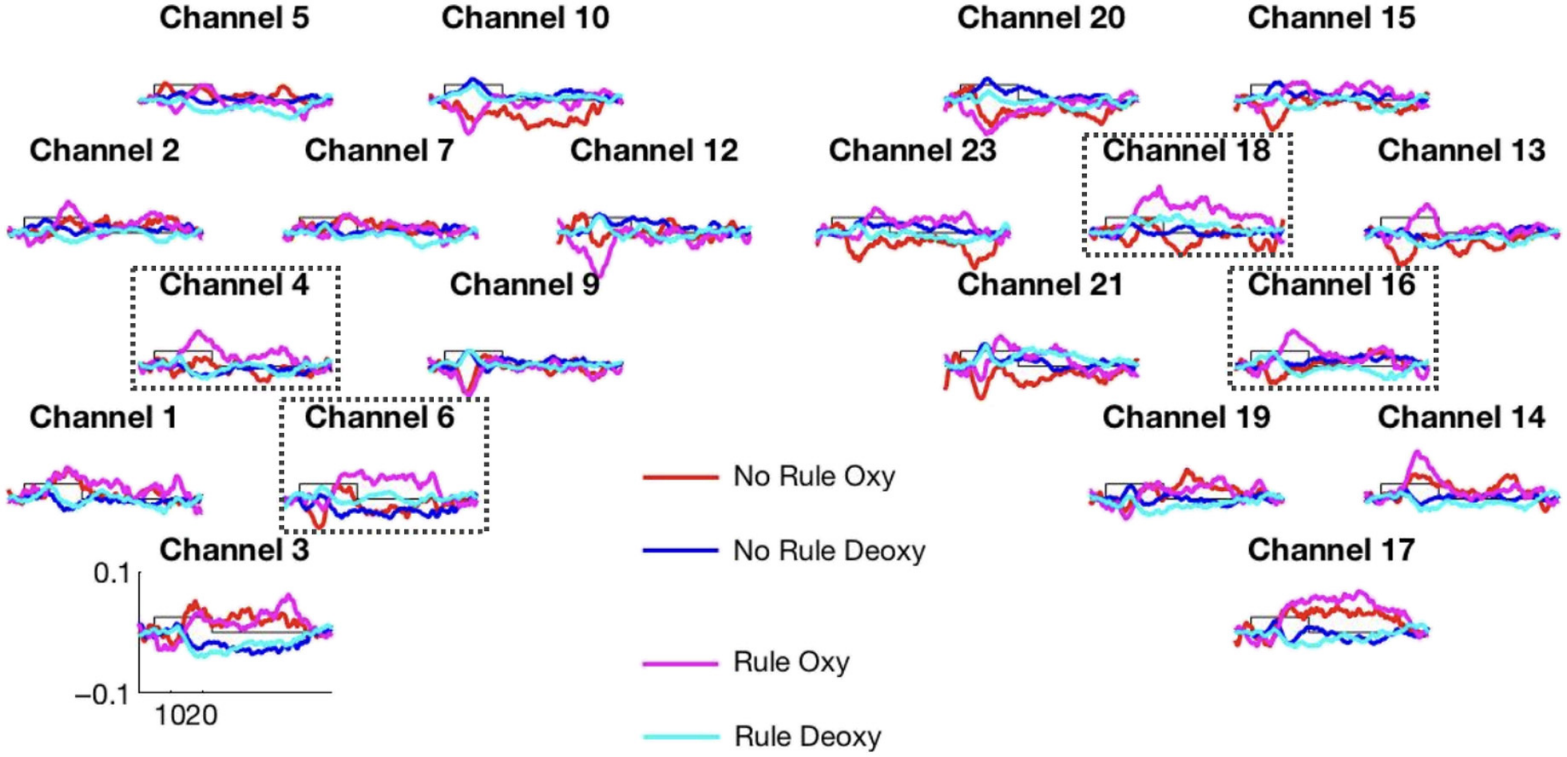
Time course plots of oxyHb and deoxyHb, averaged across all participants, shown for each fNIRS channel following the layout shown in Fig. 2 The four time traces/courses in each plot indicate oxyHb and deoxyHb for the rule and no rule conditions. Channels marked in dotted squares (4 and 6 on the left and 16 and 18 on the right) were identified as showing a significantly greater increase in oxyHb in the rule than in the no rule condition.

We preprocessed the fNIRS data using a standard and validated pipeline^13^ (see Sec. 4 for details). The overall pattern of hemoglobin changes obtained in the artificial grammar learning study is shown in Fig. 3.

We used a cluster-based permutation analysis^14^ to identify clusters of channels and time windows in which activation to the two conditions differed. The analysis over oxyHb showed greater activation to the rule than to the no rule condition in channels 4 and 6 in the left hemisphere and channels 16 and 18 in the right hemisphere. A similar analysis over deoxyHb yielded no significant findings as is commonly the case with infants.^15^^,^^16^

**Fig. 4:**
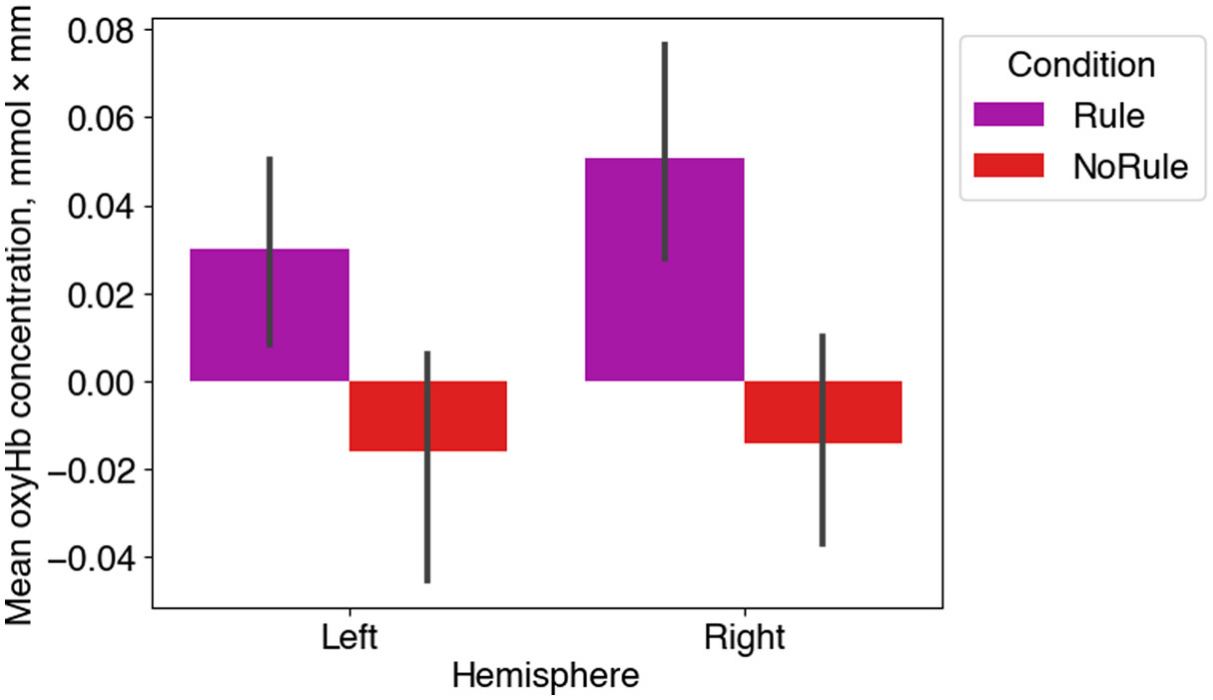
Mean oxyHb concentration for rule and no rule conditions in the left and right clusters identified by the permutation analysis, averaged across participants and trials. Error bars represent 95% confidence limits.

Figure 4 shows the average oxyHb concentration changes in the left and right clusters identified by the permutation analysis. To assess lateralization and whether there was a difference between female and male infants’ responses, we used linear mixed effects models with condition (rule/no rule), left hemisphere/right hemisphere (LH/RH), and sex (female/male) as fixed factors in the maximal model over oxyHb concentration changes. The best fitting model included condition as a fixed factor and yielded a greater increase in oxyHb in the rule than in the no rule condition (LH, mean difference, mmol×mm=0.046, t(85.5)=2.841, p=0.028; RH, mean difference, mmol×mm=0.067, t(83.3)=4.291, p=0.0003). As we did not find that sex as a factor improved our statistical models (see Data analysis section), all subsequent analyses were conducted without sex as a factor.

These results closely resemble those obtained in the previous study,^9^ with the rule condition showing greater responses in the bilateral parieto-temporal areas. As infants in the previous study were 9 months old, whereas we tested 6.5-month-olds, our results constitute even earlier evidence that as a group, very young infants can accomplish the challenging task of extracting a non-adjacent dependency when convergent statistical and prosodic cues are available. We now turn to the question of whether individual variation in brain states prior to learning can predict learning outcomes.

### 2.2 Functional Connectivity in the Period Prior to Learning

To derive a measure of brain states prior to learning, we computed functional connectivity analyses for each participant on oxyHb data during the 2-min rest period before the artificial grammar task. Specifically, we calculated correlations between all channels, following previous work.^17^^–^^19^ A notable difference between previous work with infant resting state and ours is in the filtering of the fNIRS data. Typically, studies of the “resting state” apply a different filter to fNIRS data (<0.08  Hz) compared with studies with task blocks (<0.7  Hz).^20^^–^^23^ However, as we are not making specific claims about a “resting state” that directly corresponds to the one in adults that is characterized by specific patterns of activity in the DMN, we chose to use the same, task-related filter also for the functional connectivity analysis for the period prior to learning. In addition, using a cutoff frequency of 0.7 Hz allows us to capture perceptual, attentional, memory, and other cognitive processes relevant to language, which take place at higher frequencies, because linguistic units such as sentences or words typically last between a few hundred milliseconds and a few seconds.^24^

**Fig. 5:**
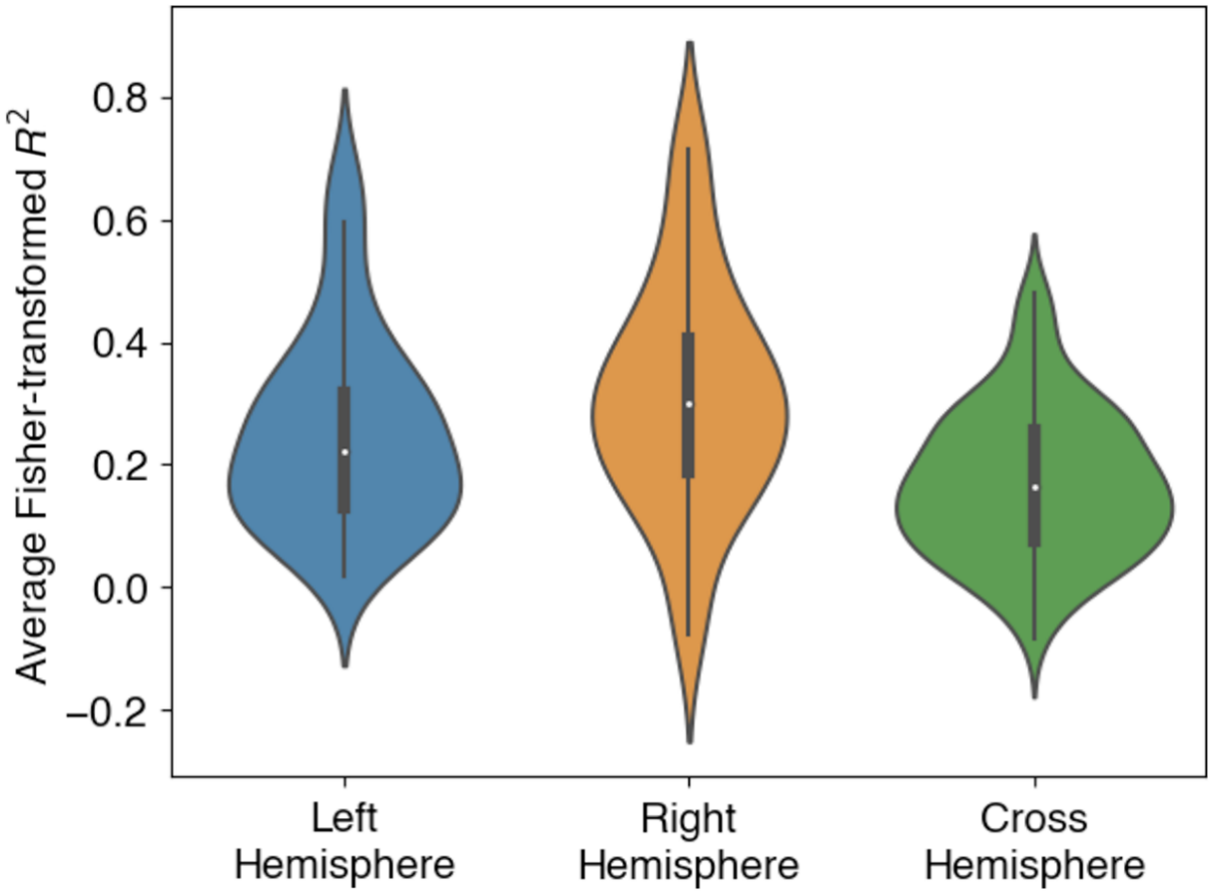
Distribution of Fisher-transformed correlation values shown separately for the left and right hemispheres and across hemispheres. The violin plots show kernel density estimates around a boxplot showing the median and quartiles of the distribution.

Figure 5 shows the distribution of connectivity scores within each hemisphere and between the two hemispheres. The correlation values were Fisher-transformed as appropriate for statistical analysis. A linear mixed effects model followed by post-hoc contrast testing showed that, although the right hemisphere had significantly higher connectivity scores than the left hemisphere (mean difference of 0.057, t(5821)=3.136, p=0.005), both hemispheres had higher connectivity values than those between hemispheres (LH, mean difference = 0.085, t(5820)=5.515, p<0.0001; RH, mean difference = 0.142, t(5820)=9.168, p<0.0001).

### 2.3 Relating Artificial Grammar Learning and Functional Connectivity Prior to Learning

**Fig. 6:**
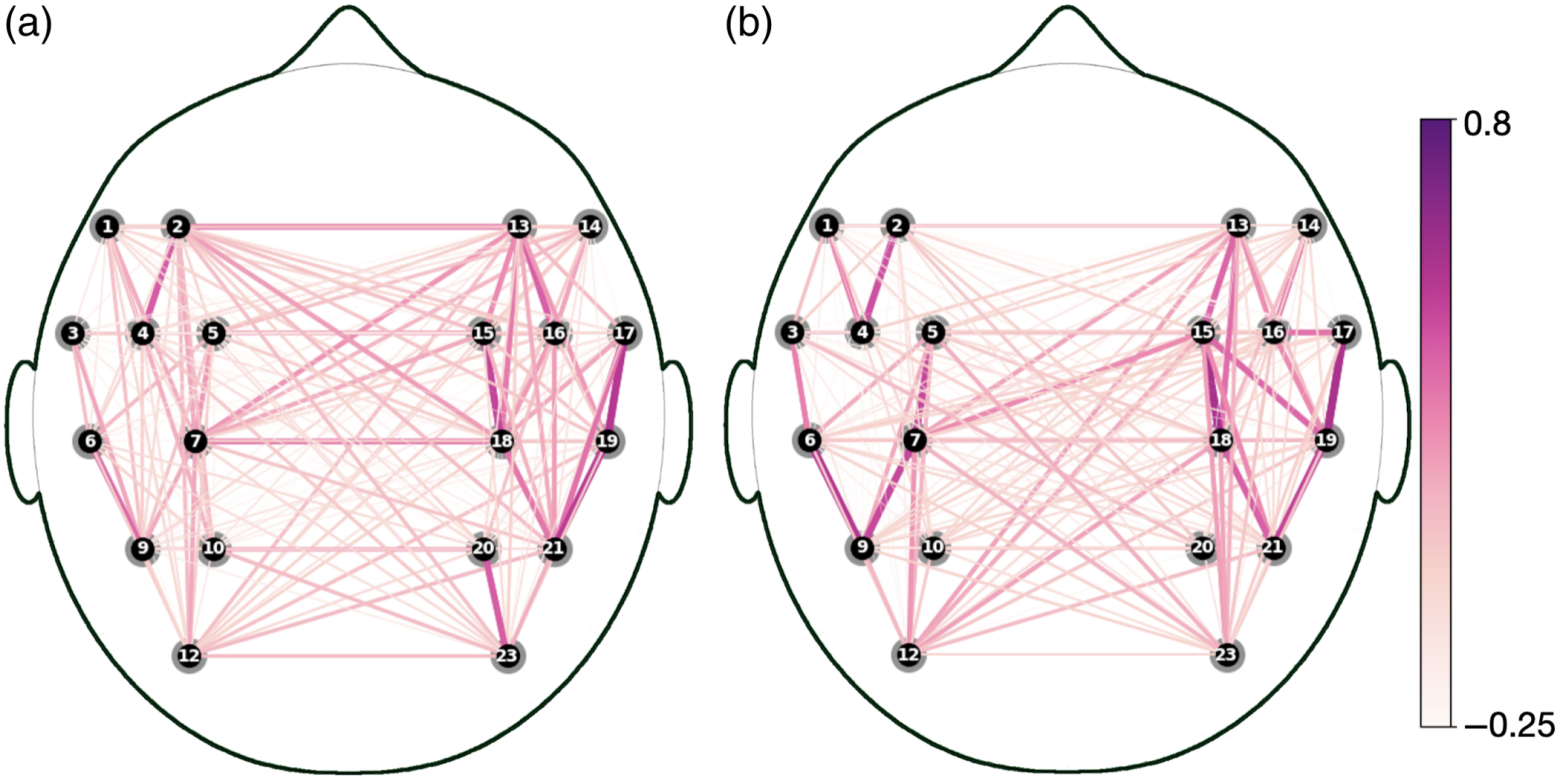
Fisher-transformed, pair-wise correlations across all 20 LH and RH channels, separated by the fNIRS index of learning. The sub-plots show average correlations for participants with below-median (a) and above-median (b) values of the fNIRS measure of learning. Line thickness and color both represent the strength of correlations between connected channels.

For illustrative purposes, we performed a median split over the infants as a function of their differential response during the artificial grammar task to see whether they showed different connectivity patterns during rest. Figures 6(a) and 6(b) show the average Fisher-transformed correlation values of functional connectivity between all channels, separately for infants with below- and above-median fNIRS index of learning, i.e., differential response in the artificial grammar task.

To investigate the relation between functional connectivity at rest and the index of learning for the left and right clusters identified by the permutation tests for the artificial grammar learning task, we used a multivariate analysis of variance (MANOVA) to determine whether the indices of learning were related to average whole-brain correlations during the resting state. We found that there was a significant effect of the resting brain state on the learning index, Pillai’s trace = 0.453, p=0.002.

**Fig. 7:**
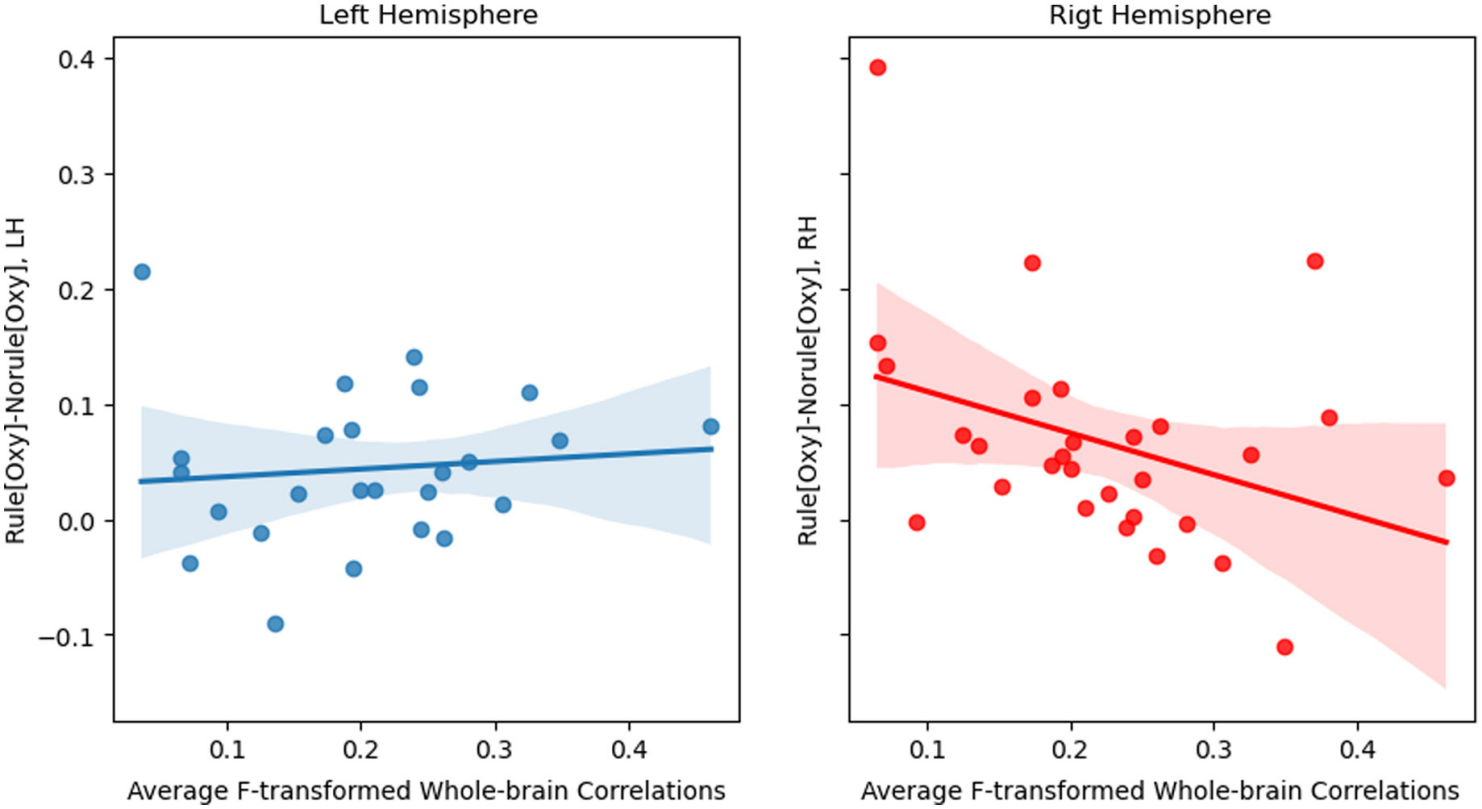
Relation between average whole-brain correlations during the rest period and the index of learning in the left and right clusters.

Given this significant result, we used robust (Huber) linear regression to look at the effect of the average whole-brain correlations on the learning index separately for the two hemispheres. We found that although this relation was not significant in the left cluster (coefficient = 0.2058, p=0.086), it was significantly negative in the right (coefficient = −0.45, p=0.002), as shown in Fig. 7.

## 3 Discussion and Conclusion

We have examined whether young infants’ brain states as measured by fNIRS functional connectivity at rest collected immediately before they were presented with a non-adjacent dependency learning task predicted the results of the learning task itself.

Our results indicate that 6.5-month-old infants already succeed in learning non-adjacent dependencies, a challenging task previously believed not to be acquired until later.^9^ Specifically, we found greater activation in response to the rule than to no rule condition in the bilateral temporo-parietal cortices.

More importantly, our findings are the first to show that the state of young infants’ brains prior to a learning experience is significantly related to a neural measure of language learning in the right but not in the left hemisphere. In particular, there was a negative correlation between whole brain connectivity and the differential response to a non-adjacent linguistic rule in the RH but not in the LH. This result indicates that rule learning is robust in the left temporal areas independently of the prior state of the infants’ brain; that is, whether the whole brain connectivity was relatively higher or lower in the period immediately before a learning experience had no bearing on the learning outcome. However, in the right hemisphere, lower average connectivity in the period immediately before a learning experience was related to better learning outcomes.

These findings differ from previous studies that typically look at the relation between differences in functional connectivity in specific (sub)networks of the brain and differences in task performance. For example, in adults, increased functional connectivity in the frontoparietal cognitive control network was associated with better memory and executive function in a longitudinal study.^25^ Similarly, in an infant longitudinal study, performance in visual versus language tasks was related to higher functional connectivity among different sets of brain areas.^26^ Here, we find that even a global metric of the brain “state” prior to a learning experience has different relations to metrics of learning outcomes in the left versus the right hemispheres. Given that increased functional connectivity generally tends to be associated with better performance (e.g., in the cited studies^25^^,^^26^) and that decreases in functional connectivity in task-relevant networks have been suggested to underlie decreased task performance, for example, in aging,^27^ our results suggest that the “contribution” of the right hemisphere for learning is better when there is less overall connectivity, which presumably includes connectivity with the LH, which is primarily important for language functions in general. These results are reminiscent of theories of language acquisition in the brain that suggest that the relative dominance of the LH in language masks the contribution of the RH.^28^ Recently, it was shown that recovery from perinatal stroke to LH language-related areas was due to homotopic areas in the RH taking on primary language processing functions.^29^ We therefore tentatively hypothesize that lower overall functional connectivity might uncover relatively independent contributions from the two hemispheres, leading to better learning outcomes.

Furthermore, the observed pattern of findings converges with existing results about early brain specialization for speech perception and language learning. As rule learning has been found to be left lateralized already at birth and^10^^,^^30^ lateralization for language increases over development,^31^ robust, state-independent learning in the LH is not surprising at 6.5 months, an age at which infants start to learn their first word forms^32^^–^^34^ and some of the basic grammatical features of their native language.^35^ This result uncovers the robust neural mechanisms explaining the implicit nature of language acquisition in young infants.

More interestingly for our research question, state-based learning was observed in the right hemisphere. Less whole-brain connectivity being a facilitator of learning suggests that the right hemisphere may be more ready to learn linguistic rules if overall networks are less active. Our paradigm is particularly well suited to identify this state-based sensitivity of the right hemisphere as the stimuli contain prosodic cues, and the right hemisphere has been shown to underlie prosodic processing from birth.^31^^,^^36^

Our results extend previous adult observations that document brain state-dependent learning, i.e., specific patterns of brain activity are related to better learning outcomes. These brain activity patterns can be constitutive or temporary. Presumably, specific constitutive brain states reflect innate and previously acquired knowledge that bootstraps learning, whereas temporary brain states reflect a combination of previously acquired knowledge and other cognitive factors such as current states of arousal or attention, which can impact acquisition. The current study was not designed to separate these two types of brain states. Future research is needed to explore the nature of brain states in greater detail.

More generally, cognitive and motor development can be seen as the creation of new (acquired) brain states, which are in turn conducive to the acquisition of other, more complex states. The infant learning literature is replete with examples where the acquisition of new behavior is dependent on the acquisition of a previous ability, as in studies under the umbrella of dynamic systems approaches.^37^ For example, 3-month-old infants with lower average velocities of limb movements show better learning of a contingent relation between moving their limbs and the movement of a toy.^38^ In a different domain, it was observed^39^ that although 9.5-month-olds do not typically use color information for object individuation, they succeed in doing so after exposure to events where the color of objects is tied to their function. In language acquisition, bilingual infant brains develop differently from their monolingual counterparts, resulting in different behaviors in novel word learning situations^40^ and cognitive gains beyond language.^41^

In the Bayesian view of the human mind/brain, even basic perception is best understood when it is properly contextualized;^42^ for example, a reddish, roundish object is more likely to be perceived as an apple in the context of an orchard in upstate New York, but a cricket ball in the context of a cricket stadium in Mumbai. At the neural level too, it has been demonstrated that conditioning-induced synaptic changes in the rat cortex depend on specific contexts—states of brain excitability.^43^ At the single-cell level, neurons in the macaque basal ganglia modulate their responses contingent on expectations, leading, for example, to faster and more accurate target recognition.^44^ The present study, in conjunction with previous adult imaging studies, provides evidence that particular brain state contexts can be more conducive to learning than other brain contexts. Across all these domains and from the neural to cognitive levels, context thus appears to play a central role.

In sum, we have shown for the first time that some parts of the infant brain, in particular the left hemisphere, are robustly and state-independently involved in language learning, whereas the involvement of other brain areas depends on infants’ brain states prior to the learning experience. These and similar findings have the potential to reveal what aspects of a child’s environment and learning context constitute teachable moments, and how interactions between the child and the environment may shape learning and development. More broadly, contextual processing might well be a general principle of cognition.

## 4 Methods

### 4.1 Participants

A total of 31 full-term French-exposed monolingual 6.5-month-olds [range 5 months;10 days to 7 months; 18 days; M age = 6 months;14 days; standard deviation (SD) = 14.7 days; 17 girls and 14 boys] were included in this study. An additional 23 infants were tested but excluded from data analysis due to fussiness and crying (n=9) and insufficient valid data (n=13) or because the headgear was too small (n=1). All data quality assessments and rejections were conducted in batches, prior to statistical analysis.

This study was approved by the CERES ethics board (Université Paris Cité, France), and all methods were carried out in accordance with relevant guidelines and regulations. Parents/legal guardians gave informed consent before participation.

### 4.2 Stimuli

Auditory stimuli followed previous rule learning studies using fNIRS with infants^30^ and were the same as those used in a previous study.^9^ Stimuli were generated using the fr4 French female voice diphone database of MBROLA.^45^ All syllables were consonant-vowel, with a duration of 120 ms for consonants and 150 ms for vowels. The pitch of the initial and final syllables of each of the tri-syllabic nonce “words” was set to 220 Hz, whereas the middle syllable was set to 200 Hz.

Auditory stimuli in the rule blocks consisted of 72 trisyllabic “words” following an AxB rule, that is, there was a systematic dependency between the identity of the first and the last syllable, whereas the middle syllable was allowed to vary. There were a total of two AxB “families” (*pe* x *bu*, *to* x *shi*), and the middle syllable was randomly chosen from a set of 18 syllables (*ba, bo, di, du, fe, go, ke, lo, la, mu, me, na, pi, ta, she, shu, zo, zi*).

Trisyllabic sequences in the no rule blocks consisted of the same set of syllables as in the rule blocks, with the same overall frequencies, but now arranged into triplets that did not have a consistent AxB structure. These trisyllables were of the form ABx, AxB, BAx, BxA, xAB, and xBA.

### 4.3 Procedure

Infants were individually tested in a quiet room with the lights dimmed. Infants sat on their caregivers’ laps, around 80 cm from a screen on which a silent animation video played for the duration of the study. Caregivers listened to masking music over tight-fitting headphones.

All experimental stimuli were presented via E-Prime software (Psychology Software Tools, Pittsburgh, Pennsylvania, United States). Stimuli were presented in eight blocks of 18 trisyllables each, half of which were rule blocks, whereas the other half were non rule blocks (see Fig. 1). No more than two consecutive blocks of the same type were allowed. In each block, each trisyllabic word was presented without a pause between the three constituent syllables, and the inter-stimulus interval between one trisyllable and the next was randomly chosen to be 0.5 or 1.5 s. There was a pause of either 25 or 35 s between one block and the next. Blocks were pseudorandomized and counterbalanced across participants. The entire experiment lasted 8.34 min.

fNIRS data were collected via a NIRx NIRScout machine with eight sources and eight detectors organized into 20 channels, 10 on the left hemisphere and 10 on the right. Source-detector separations were 3 cm, and changes in light absorbance at 760 and 850 nm were collected at a sampling rate of 15.625 Hz. Localization analyses indicated that channels 2 and 13 are located over the frontal lobe; channels 1, 3, 6, 9, 14, 17, 19, and 21 are placed over the temporal lobe; channels 10, 12, 20, and 23 query the parietal lobe; and channels 1, 4, 5, 7, 15, 16, and 18 span two lobes.^9^ Prior to the start of the experiment proper, 2 min of fNIRS data was recorded in silence.

### 4.4 fNIRS Data Preprocessing

fNIRS preprocessing followed the same pipeline as in previous infant fNIRS studies.^9^^,^^13^ Briefly, intensities were converted to relative changes in oxygenated (oxyHb) and deoxygenated (deoxyHb) hemoglobin, a band pass filter between 0.01 and 0.7 Hz was used to eliminate slow signal drift as well as physiological variations due to heartbeat and respiration. Large and rapid changes in intensity (>0.1  mol×mm over two adjacent samples), indicative of movement artifacts, were marked and rejected in channel-block pairs.

The data for each of the remaining artifact-free blocks were sliced to a time window starting from 5 s before stimulus onset to 20 s from stimulus offset (to allow for a return of the hemodynamic response to baseline). A linear fit between the mean of the first 5 s and the last 5 s of this window was removed to detrend the data. Only participants with at least 50% artifact-free blocks were entered into the final analysis.

Separately, the oxyHb and deoxyHb signals for the 2 min of rest prior to the artificial grammar task were extracted, similarly preprocessed, and saved for further connectivity analysis. The oxyHb and deoxyHb values for the rest period in the final set of participants were artifact-free (by the criteria defined above), except for two channels for one participant; these two channels were excluded from the connectivity analysis.

### 4.5 Statistical Analysis

To establish region of interest (ROI) and time windows of interest in a data-driven way, we performed cluster-based nonparametric permutation tests ^14^ in MATLAB on the fNIRS data from the artificial grammar task as in earlier studies.^9^ Permutation analyses were carried out separately for oxyHb and deoxyHb. Briefly, this method finds spatially adjacent channels in which significant differences, as determined by two-tailed, paired sample t-tests between the conditions being compared, are observed in temporally adjacent samples, thus avoiding the issue of multiple comparisons. We ran 1000 permutations to establish the chance distribution. As there were no significant windows identified by the permutation analysis for deoxyHb, we did not consider deoxyHb further.

All further data manipulations, analyses, and figure creations were carried out in Python 3 using standard libraries pandas, numpy, matplotlib, and Seaborn and in R, using the lmer and emmeans libraries.

To compare differences in neural responses to rule and no rule blocks, the responses in the ROIs and time windows identified by the permutation analysis were averaged, separately for the LH and RH, to obtain two measures (oxyHb for LH and RH) per baby. We first compared linear mixed effects models with participants as a random effect, hemisphere, and condition as fixed effects, with and without sex as a fixed effect, starting from the maximal model:

(MeanOxy Hemisphere*Condition*Sex+(1+Condition|BabyFilename),data=fNIRSdata),

all the way through to the simplest model:

(MeanOxy Hemisphere*Condition+(1+Condition|BabyFilename),data=fNIRSdata).

We compared these models in an analysis of variance (ANOVA). Although the differences between models were not significant, models without sex as a factor had lower AIC/BIC values, with the minimal model having the lowest Akaika Information Criterion (AIC)/Bayesian Information Criterion (BIC). We therefore report findings from only the minimal model.

Relevant post-hoc comparisons by hemisphere × condition were carried out on the minimal linear mixed-effects model:

pairs(emmeans(model,Hemisphere*Condition))

### 4.6 fNIRS index of learning

To capture a single hemodynamic index of learning in the artificial grammar task, oxyHb and deoxyHb data were first extracted from those channels and time regions that the permutation analysis identified in a given hemisphere. For all channel and time window combinations, oxyHb was higher in the rule than in the no rule condition (see Fig. 4). So, we defined the index of learning for oxyHb as

$${\text{index}}^{\mathrm{Oxy}}=oxyHb_{\text{Rule}}-oxyHb_{\mathrm{No}\text{Rule}}.$$

For each infant, we computed one ${\text{index}}^{\mathrm{Oxy}}$ for the left hemisphere and one for the right.

### 4.7 Functional connectivity analysis

One way to define resting-state functional connectivity over infant NIRS data is to calculate pairwise correlations between all channels.^19^ To capture the brain “state” in a singular metric, we first computed the correlation coefficients between all channel pairs from the 2 min resting period data. These were then Fisher (arctanh) transformed and averaged to give a single value for each infant, for each hemisphere separately, as well as for the whole brain. Statistical analyses were carried out using the statsmodel Python package.^46^

## Data Availability

Given the sensitive nature of the biomedical data involved, data are available from M.S. or J.G. upon reasonable request. Materials are available upon request from J.G.
